# Hypothetical endogenous SIV-like antigens in Mauritian cynomolgus macaques

**DOI:** 10.6026/97320630014048

**Published:** 2018-02-28

**Authors:** Hongzhao Li, Lin Li, Lewis R Liu, Robert W Omange, Nikki Toledo, Mohammad Abul Kashem, Yan Hai, Binhua Liang, Francis A Plummer, Ma Luo

**Affiliations:** 1 Department of Medical Microbiology and Infectious Diseases, University of Manitoba, Winnipeg, MB R3E 0J9, Canada;; 2 National Microbiology Laboratory, Public Health Agency of Canada, Winnipeg, MB R3E 3L5, Canada;; 3 Department of Biochemistry and Medical Genetics, University of Manitoba, Winnipeg, MB R3E 3N4, Canada;

**Keywords:** SIV, Mauritian cynomolgus macaques, HIV, vaccine, protease cleavage sites (PCS), non-PCS, database

## Abstract

Simian immunodeficiency virus (SIV) infection of Mauritian cynomolgus macaques (MCMs) is an increasingly important nonhuman primate model for HIV vaccine research. We previously reported that in MCMs anti-SIV antibodies can be naturally developed without exogenous infection or vaccination, and that a vaccine targeting SIV protease cleavage sites (PCS) can cross-induce antibodies to non-PCS SIV antigens. We speculate that this is potentially caused by the existence of endogenous SIV-like antigens. External stimuli (such as environmental factors and vaccination) may induce expression of endogenous SIV-like antigens to elicit these antibodies. Database and mass spectrometry analyses were conducted to search for such antigens. We identified endogenous SIV-like DNA sequences in cynomolgus macaque genome and non-PCS peptide homologous to SIV Env protein in PBMCs of a PCS-vaccinated monkey. Our preliminary insights suggest that endogenous SIV-like antigens may be one of the possible reasons for the natural and cross-inducible SIV antibodies in MCMs.

## Background

Simian immunodeficiency virus (SIV) infection of nonhuman primates (NHPs) is currently the best animal model to test HIV vaccine strategies or study HIV pathogenesis [1, 2, 3, 4, 5, 6, 7, 8, 9, 10, 11, 12, 13, 14]. Traditionally, rhesus macaques (Macaca mulatta) are the favorite choice among NHPs in HIV vaccine studies [1, 2, 3, 4, 5, 6, 7]. However, the availability of rhesus macaques has been greatly reduced due to a ban of their export from India and most other south Asian countries [6, 15]. Cynomolgus macaques (Macaca fascicularis) have become by far the most internationally traded NHP for laboratory experiments [6]. The largest laboratory supply of cynomolgus macaques is available from the island of Mauritius. The Mauritian cynomolgus macaques (MCMs) descended from a small group of founder animals and are characterized by high genetic homogeneity with much simpler major histocompatibility complex (MHC) haplotypes and fewer alleles [6, 7, 13, 16, 17, 18]. This animal model has lower variability between animals and thus reduces the number of animals needed to achieve statistical power, making them practical for HIV vaccine studies [7].

During HIV or SIV replication, each of the 12-protease cleavage reactions is essential for the production of a functional viral particle [19]. A novel vaccine strategy targeting the protease cleavage sites (PCS) has been suggested by our studies [14, 19, 20] and is being evaluated using MCM SIV infection model [12]. Commonly, vaccine studies are carried out in specific pathogenfree animals to rule out the impact of on-going infection or preexisting immune responses in order to solely evaluate the vaccine efficacy absent of confounding variables. In a pilot study, we used PCS peptide antigens (along with non-PCS peptides) to screen for potentially pre-existing natural antibody responses in MCMs [12], while, unlike in several other NHP species [21], no natural immune response screen study had been reported in MCMs. Specifically, the SIV antigens were twelve 20mer peptides overlapping the twelve PCS (-10/+10) and three non- PCS Gag or Env peptides of SIVmac239 [22, 23, 24]. In some MCMs antibodies to these antigens were found to be very high in the absence of exogenous infection or vaccination [12]. We also observed that immunization of MCMs with PCS vaccine not only elicited antibodies to the PCS peptides, but also cross-induced antibodies to non-PCS peptides, while the non-PCS peptides share no sequence homology with the PCS peptides [12], suggesting that the PCS vaccine could elicit off-target immune response [25] targeting SIV antigens that are not in the vaccine. Since understanding natural and vaccine cross-inducible immune responses is expected to provide important information and clues for vaccine development [21, 25], we report here a possible reason that may contribute to the existence of these anti-SIV antibodies in MCMs.

## Methodology

### Humane care guidelines

The MCM plasma sample used in the current study was collected in our recently published animal work. The human care guidelines were described in detail in that publication [12].

### The PCS and non-PCS SIV peptides

These SIV peptides, derived from SIVmac239 [22, 23, 24], are twelve 20mer peptides overlapping the twelve protease cleavage sites (- 10/+10), named as PCS1 through PCS12, and three non-PCS Gag or Env peptides, named SIVgag, SIVenv1 and SIVenv2. The sequences of these peptides were listed in the recent publication [12]. They were confirmed to be specific for SIV by NCBI protein BLAST and conserved among multiple SIV strains. No sequence homology was shared between PCS versus non-PCS peptides [12].

### Identification of SIV-like sequences in the cynomolgus macaque genome

SIV peptide coding sequences were searched in cynomolgus macaque whole genome shotgun sequences for each chromosome using NCBI nucleotide BLAST (BLASTN Suite).

### Enrichment of SIV peptide-specific antibodies

This was performed as previously described [12].

### Mass spectrometry identification of endogenous SIV-like antigen

SIVenv2 peptide-specific antibodies enriched from monkey plasma were incubated with Pierce protein A/G agarose beads (ThermoFisher Scientific, Catalog 20422) and further cross-linked using dimethyl pimelimidate (DMP) (Sigma Aldrich, St. Louis, MO; Catalog D8388). Potential SIVenv2-like antigen was enriched from monkey PBMC lysates by immunoprecipitation using these SIVenv2 antibody-coupled beads and then analyzed by 2D LCMS/ MS as below.

The protein/peptide samples were digested with trypsin in solution. Briefly the samples were concentrated to near-dryness (1-5μl) using a vacuum centrifuge (Savant Speed/Vac Concentrator, Thermo Fisher Scientific). The samples were resuspended in 25μl of 50mM HEPES pH 8.3 and mixed for 30 minutes. 2μl of 50mM DTT (Sigma) in 100mM ammonium bicarbonate (AB, Fisher Scientific) was added and the samples mixed and incubated at 37°C for 1 hour. 1μl of 200mM Iodoacetamide (Sigma) in AB, was added, the samples mixed and incubated at room temperature (in the dark) for 10 minutes. Trypsin (Pierce, Thermo Scientific, 2.5μg per 100μg protein) was added and the samples were mixed and incubated at 37°C overnight in a humidified chamber. After digestion, the tryptic peptides were collected and concentrated to near-dryness (1-5μl) using a vacuum centrifuge and re-suspended in MS buffer A (below) for further analysis.

Samples were analyzed using a nano-flow Easy nLC I connected in-line to an LTQ Orbitrap XL mass spectrometer with a nanoelectrospray ion source at 2.1 kV (ThermoFisher Scientific, San Jose, CA). The peptide fractions were loaded (5μl) onto a C18-reversed phase trap column (3 cm long, 100 μm inner diameter, 5 μm particles) with 100% buffer A (2% acetonitrile, 0.1% formic acid) for a total volume of 30 μl, and then separated on a C18-reversed phase column (15 cm long, 75μm inner diameter, 2.4μm particles). Peptides were eluted using a linear gradient of 2-35% buffer B (98% acetonitrile, 0.1% formic acid) over 40 min at a constant flow rate of 250nl/min. Total LC/MS/MS run-time was 80 minutes, including the loading, linear gradient, column wash at 95% buffer B, and the equilibration.

Data was acquired using a data-dependent method, dynamically choosing the top 5 abundant precursor ions from each survey scan for isolation in the LTQ (2.0 m/z isolation width) and fragmentation by CID (35% normalized collision energy, with 30 ms activation time). The survey scans were acquired in the Orbitrap over m/z 300-1700 with a target resolution of 60000 at m/z 400, and the subsequent fragment ion scans were acquired in the LTQ iontrap. The lower threshold for selecting a precursor ion for fragmentation was 1000 ions. Dynamic exclusion was enabled using a list size of 500 features, a m/z tolerance of 15ppm, a repeat count of 1, a repeat duration of 30s, and an exclusion duration of 60s, with early expiration disabled.

Raw files were loaded into PEAKS Studio v 7.5 (Bioinformatics Solutions Inc., Waterloo, ON, Canada). Data were refined without merging, with precursors corrected and only spectra with a charge of +2 to +6 being accepted with a filter quality of 0.65 or better. De novo searching was performed on the peak list using the following search parameters: Carbamidomethylation was selected as a fixed modification, oxidation as a variable modification, fragment ion mass tolerance of 0.5 Da, parent ion tolerance of 10 ppm, and trypsin as the enzyme. De novo list was exported with an ALC(%) set to 50.

For homology search, peptides identified by mass spectrometry were compared with the Non-redundant UniProtKB/SwissProt sequences database (Updated on 28 May 2016) using the Biopython [26] module 'NcbiblastpCommandline' as a wrapper for the offline command-line tool Blast+ [27]. The search was conducted with BLASTP algorithm (version 2.2.28+) [28, 29]using PAM30 scoring matrix with 'blastp-short' task settings but with composition-based score turned off. (Specific settings: matrix=PAM30, comp_based_stats=0, ungapped=True, seg=no, neighboring word threshold=16, window for multiple hits = 15). The resultant hit list was screened for the term "SIV" by parsing in python, and then manually verified.

## Results

### A proposed model: Endogenous SIV-like antigens may contribute to natural or vaccine cross-inducible anti-SIV antibodies

**Figure 1:**
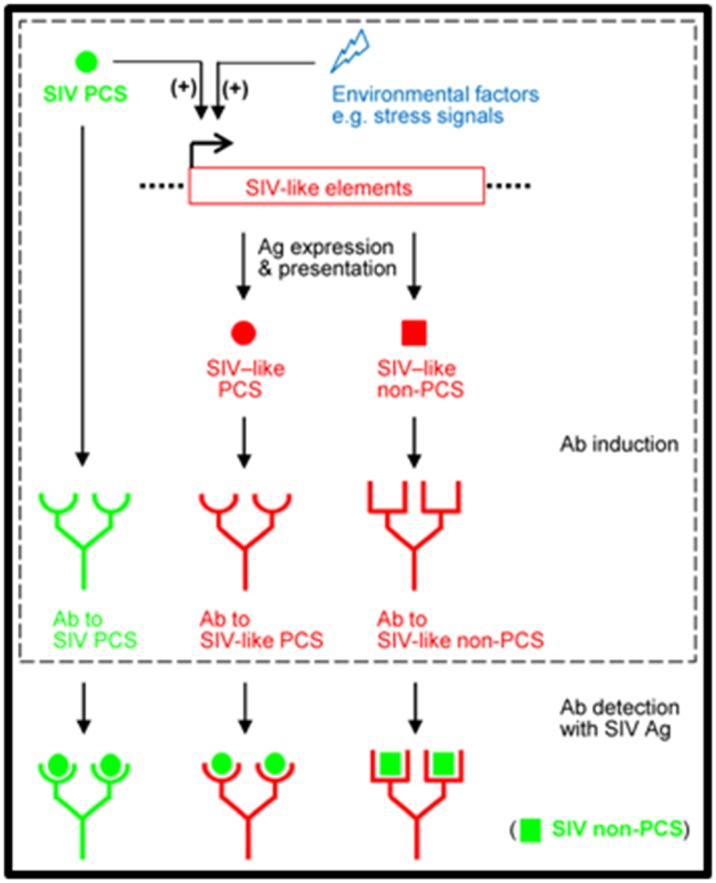
Hypothetical model: Endogenous SIV-like antigens may contribute to natural or PCS vaccination-induced SIV antibodies. Endogenous SIV-like retroviral sequences in Mauritian cynomolgus macaques encode PCS-like and non-PCS-like peptides. Environmental factors such as stress or PCS vaccination activates the expression of these antigens, subsequently leading to the induction of host antibodies to both PCS-like and non-PCSlike antigens.

We previously conducted a pilot study to test a novel HIV vaccine strategy, which targets the twelve viral protease cleavage sites (PCS), using the MCM SIV infection model [12]. SIV peptides based on the sequences of the PCS sites (PCS peptides), along with non-PCS peptides, were used as antigens to screen SIV antibody-negative animals for vaccination experiments. PCS peptides were also delivered as immunogens in the form of recombinant vesicular stomatitis viruses and nanoparticles (the PCS vaccine). We detected high-level natural antibodies to PCS and non-PCS SIV peptides [12]. The source of antigens that induced natural SIV antibodies in these monkeys is unknown, since they were healthy animals obtained from SIV-free breeding colony and without any on-going SIV infection. An important clue was suggested by the vaccination experiment, in which the PCS vaccine surprisingly induced antibodies to non-PCS antigens, although the non-PCS antigens share no sequence homology with the PCS antigens [12]. One possible explanation for the cross-induction is that the SIV PCS peptides might, by unknown mechanism(s), have stimulated the production of endogenous SIV-like antigens that contain peptide sequences antigenically similar to non-PCS SIV peptides (Figure 1).

It was reported that expression of endogenous retroviral elements [30, 31, 32] can be triggered by external stimuli such as HIV infection in humans [30, 33, 34, 35, 36, 37, 38, 39, 40, 41, 42] and that endogenous retroviral antigens present in normal baboon reproductive tissues can be cross-recognized by HIV/SIV antibodies [43, 44]. While other possibilities may exist, we speculate that external stimuli such as environmental factors and the PCS vaccine might activate endogenous retrovirus (ERV), leading to the expression of endogenous SIV-like antigens and induction of antibody responses to the resulting viral antigens (Figure 1).

### Identification of potential endogenous SIV-like antigens

**Figure 2:**
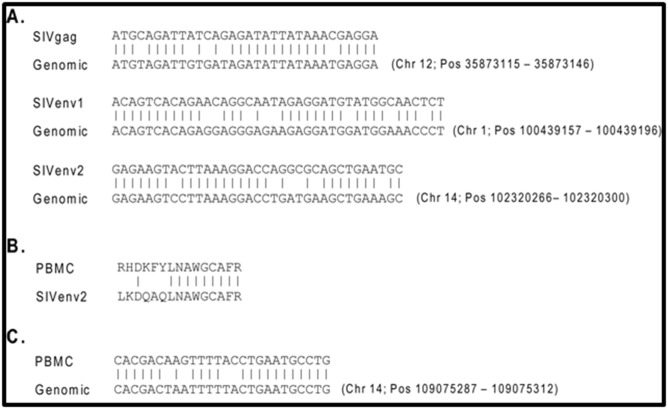
Identification of potential endogenous SIV-like antigen: (A) Coding sequences of the three SIV non-PCS peptides were searched against cynomolgus macaque genomic shotgun sequences. Numerous genomic sequences similar to each of the non-PCS sequences were found in multiple chromosomes. One representative example is shown for each non-PCS. Homologous regions are indicated by lines connecting identical nucleotides. The chromosome (Chr) numbers and positions (Pos) of the genomic sequences are listed. (B) A peptide antigen with SIV-like sequence was identified from cynomolgus macaque peripheral blood mononuclear cells (PBMC). This was based on mass spectrometry analysis of immunoprecipitation-enriched PBMC antigens using non-PCS peptide SIVenv2-specific antibodies that were affinity-purified from PCS-vaccinated monkey plasma. See Materials and Methods for experimental details. Homologous regions between the sequence of the identified PBMC antigen peptide and that of SIVenv2 are indicated by lines connecting identical nucleotides. (C) The DNA sequence of the SIVenv2-like peptide identified in PBMC was BLAST-searched and aligned to the MCM genome. Homologous regions are indicated by lines connecting identical nucleotides. The chromosome (Chr) number and position (Pos) of the genomic sequence are listed.

We BLAST searched cynomolgus macaque genomic shotgun sequences for the three non-PCS peptide-coding sequences and identified SIV non-PCS-like sequences on multiple chromosomes (Figure 2A). To examine the expression of endogenous SIV-like antigen on the protein level, we coupled a non-PCS peptide, SIVenv2, to affinity purification columns to enrich its specific antibodies from a PCS-vaccinated monkey. The purified antibodies were then used to immuno-precipitate antigens from peripheral blood mononuclear cells (PBMC) lysates of the PCSvaccinated monkey. The enriched antigens were then analyzed by Mass Spectrometry and searched in sequence databases. A SIVenv2-like peptide sequence was identified from the monkey PBMC by this procedure (Figure 2B). We then performed a BLAST search for the DNA sequence of the SIVenv2-like peptide identified in PBMC and found that it was aligned to the MCM genome (Figure 2C). These results are consistent with the hypothesis that activation of dormant SIV-like antigens in MCMs by external stimuli might be one of the potential mechanisms for generation of SIV antibodies (natural or vaccine cross-induced) (Figure 1). This possibility will need to be validated by future investigations.

## Discussion

Based on the presence of natural and vaccine cross-inducible anti- SIV antibodies as described in the background, we speculated that endogenous dormant SIV-like antigens could be expressed upon activation by environmental factors or vaccination and subsequently induce antibody responses. A broad body of literature showed the presence of endogenous retroviral sequences in primate genomes [30, 31, 32, 33, 43, 44, 45, 46, 47, 48, 49, 50]. A vast array of germline-integrated retroviruses, formed during primate evolution and transmitted vertically (from parents to offspring) in Mendelian manner, are classically defined as endogenous retroviruses. As remnants of ancient retroviral infections, these are commonly structurally incomplete with mutations and deletions. However, some of them retain the potential to express viral proteins or peptides in response to cellular stimuli or stress [30, 33, 34, 35, 36, 37, 38, 39]. For example, HIV infection activates human ERVs including HERV-K and LINE-1 [33, 34, 35, 36, 37, 38, 39, 40, 41, 42, 51]. It has also been proposed that other than inherited, dormant proviruses can also be acquired through previous infection by retroviruses from the hosts' living environments and carried by the hosts without viral replication and subsequent induction of host immune responses [30, 48]. For simplicity, these and the classical endogenous retroviruses are collectively referred to as "endogenous" retroviruses (ERV) in the context of this work, considering their nature of being dormant "intrinsic" residents within the hosts. Importantly, studies in normal baboons suggested antigenic similarity of ERVs with HIV or SIV [43, 44]. We speculate that activation of SIV-like ERV in MCMs by environmental factors (such as stress or infection) or vaccination might be an endogenous source of antigens that induce anti-SIV antibodies. This hypothesis seems to be supported by our identification of SIV-like sequences in the genome and SIV-like peptide in PBMC of these animals.

Given SIV-like ERVs as a possible source of antigens that might induce anti-SIV antibodies, their specific identification would be an interesting future direction. In addition, apart from their possible activation by SIV PCS peptides in immunization experiments, in the context of their potential involvement in natural antibody induction, the stimuli that might trigger their activation remain to be explored. ERVs can be activated by a variety of stress signals from the surroundings of the cell, tissue, organ or system levels that alter their transcription environment or epigenetic status, such as infection, injury, oxidative stress and psychological stress [30].
